# The Anti-Inflammatory Effect of Preventive Intervention with Ketogenic Diet Mediated by the Histone Acetylation of *mGluR5* Promotor Region in Rat Parkinson's Disease Model: A Dual-Tracer PET Study

**DOI:** 10.1155/2022/3506213

**Published:** 2022-09-05

**Authors:** Yuankai Zhu, Xiangyu Tang, Zhaoting Cheng, Qingjian Dong, Ge Ruan

**Affiliations:** ^1^Department of Nuclear Medicine, Tongji Hospital, Tongji Medical College, Huazhong University of Science and Technology, Wuhan, China; ^2^Department of Radiology, Tongji Hospital, Tongji Medical College, Huazhong University of Science and Technology, Wuhan, China; ^3^Department of Radiology, Hospital, Hubei University, Wuhan, China

## Abstract

**Materials and Methods:**

The neuroprotective effect of ketosis state prior to the onset of PD (preventive KD, KDp) was compared with that receiving KD after the onset (therapeutic KD, KDt) in the lipopolysaccharide- (LPS-) induced rat PD model. A total of 100 rats were randomly assigned to the following 4 groups: sham, LPS, LPS + KDp, and LPS + KDt groups.

**Results:**

Significant dopamine deficient behaviors (rotational behavior and contralateral forelimb akinesia), upregulation of proinflammatory mediators (TNF-*α*, IL-1*β*, and IL-6), loss of dopaminergic neurons, reduction of mGluR5^+^ microglia cells, increase of TSPO^+^ microglia cells, reduction of H3K9 acetylation in the *mGluR5* promoter region and mGluR5 mRNA expression, and decline in the phosphorylation levels of Akt/GSK-3*β*/CREB pathway were observed after the intervention of LPS (*P* < 0.01). TSPO and DAT PET imaging revealed the increased uptake of ^18^F-DPA-714 in substantia nigra and decreased uptake of ^18^F-FP-CIT in substantia nigra and striatum in LPS-treated rats (*P* < 0.001). These impairments were alleviated by the dietary intervention of KD, especially with the strategy of KDp (*P* < 0.05).

**Conclusions:**

The anti-inflammatory effect of KD on PD was supposed to be related to the modulation of Akt/GSK-3*β*/CREB signaling pathway mediated by the histone acetylation of *mGluR5* promotor region. The KD intervention should be initiated prior to the PD onset in high-risk population to achieve a more favorable outcome.

## 1. Introduction

Parkinson's disease (PD) affects more than 6 million patients worldwide [1, 2]. This neurodegenerative disease was the fastest growing neurological disorder in prevalence. The disability-adjusted life-years and death rates for PD also showed an increasing trend. Apart from the cardinal motor symptoms induced by the striatal dopaminergic deficiency, the impact from various concurrent nonmotor symptoms has been increasingly recognized [3, 4]. Though the primary hallmark of PD is definitely the reduction of dopaminergic neurons in the substantia nigra (SN) located at mesencephalon, reversing its progression or prevention is still a challenge [5]. This dilemma should be attributed to the poor understanding of the complex etiopathology related to PD.

A complicated network of crosstalk between neurons, glia, and immune cells plays a crucial role in the onset and progression of PD [6]. Reactive microglia have been observed in the SN of PD subjects, suggesting the involvement of neuroinflammation [7]. Therefore, apart from the current treatment strategy by maintaining the dopamine balance, anti-inflammatory therapy is a promising field to explore novel neuroprotective strategy for PD. The expression of Translocator Protein 18 kDa (TSPO), first described as the peripheral benzodiazepine receptor, was considered to be upregulated in the activated microglia [8]. This biomarker for brain neuroinflammation can be accessed by using TSPO positron emission tomography (PET) neuroimaging [9]. Compared with the radiotracer of ^11^C-PK11195, ^18^F-DPA-714 showed better imaging performance on affinity, bioavailability, and signal-to-noise ratio (SNR) [10, 11]. Moreover, the noninvasive neuroreceptor PET imaging also allowed the *in vivo* evaluation of the aberrant nigrostriatal dopamine transporter (DAT) function, which was one of the most prominent and sensitive biomarkers in the early stage of PD [12].

Ketogenic diet (KD), characterized by a high-fat, adequate protein, and low-carbohydrate content, has been employed for the treatment of epilepsy, and even for suppressing the superrefractory status epilepticus [13]. In view of the feasibility, safety, and efficacy, its application in PD was also investigated [14]. PD subjects following KD rules showed significant improvements in both motor and non-motor symptoms [15–18]. Despite the fact that the precise mechanism for the neuroprotective actions on PD remains controversial [19], the KD could exert anti-inflammatory effects mediated by multiple approaches [20]. In addition to the direct inhibition of releasing proinflammatory cytokines induced by NOD-, LRR-, and pyrin domain-containing 3 (NLRP3) inflammasome assembly, the anti-inflammatory effect of PD can also be partly explained by the epigenetic mechanisms, for example, histone acetylation [21, 22]. The determination of corresponding signal pathways mediated by histone acetylation in microglia under KD state would be critical for developing novel targeted interventions. Besides, to the best of our knowledge, the preventive effect of KD on the PD onset has not been elucidated yet.

The current study intended to explore the epigenetic anti-inflammatory mechanism of KD for the neuroprotective actions on PD, via *in vivo* TSPO and DAT dual-tracer PET imaging combined with *in vitro* molecular biological detection in the rat model of PD. In addition, the neuroprotective effect of ketosis state prior to the onset of PD (preventive KD, KDp) was compared with that receiving KD after the onset (therapeutic KD, KDt).

## 2. Materials and Methods

Four-week-old male Sprague–Dawley rats were group-housed under standard conditions on a 12-hour light/dark cycle. All rats had free access to food and water. A total of 100 rats were randomly assigned to the following 4 groups: sham, lipopolysaccharide (LPS), LPS + KDp, and LPS + KDt groups. Stereotactic injection was performed for all rats 4 weeks later. Behavioral tests, PET imaging, and *in vitro* experiments were studied at another 4 weeks after the operation of stereotactic injection. The sham and LPS groups were fed with control diet (CD), containing 15% protein, 65% carbohydrates, and 20% fat based on energy source. The LPS + KDp group was fed with KD, containing 15% protein, <1% carbohydrates, and 89% fat. The LPS + KDt group was fed with CD for 4 weeks initially, but with KD after the LPS injection.

### 2.1. LPS-Induced PD Model

After being anesthetized with sodium pentobarbital, animals were placed in a stereotaxic instrument. For the LPS, LPS + KDp, and LPS + KDt groups, LPS solution (5 *μ*g/2 *μ*l; L2880, Sigma-Aldrich) was injected into the substantia nigra (SN) pars compacta with a 28 G Hamilton syringe. The stereotaxic coordinates were as follows: anteroposterior, −5.2 mm from the bregma; mediolateral, −2.0 mm from the midline; and dorsoventral, 7.8 mm below the dura. At the end of the infusion, the syringe was left implanted for an additional 5 min and slowly retracted. Rats in sham group were conducted with the same procedure, except the 2 *μ*l 0.9% saline rather than LPS solution that was injected into the SN.

### 2.2. Behavioral Tests

In hemi-Parkinsonian rats, sensitized striatum should be more prone to be stimulated by apomorphine (APO), resulting involuntary rotation. The APO-induced rotation test was performed 4 weeks after stereotactic injection (*n* = 5 for each group) [23]. All rats were injected with 1 mg/kg of APO s.c., and the number of the turns was recorded for 30 min.

The cylinder test was performed in these rats as well, to compare the spontaneous use of each single forelimb [24]. The number of touching the cylinder wall with each forelimb was appraised separately for 5 min. Based on the total touches, the proportion of using contralateral limb was determined and compared among different groups.

### 2.3. Small Animal Brain PET Scan and Image Analysis

The syntheses of ^18^F-DPA-714 and ^18^F-FP-CIT were conducted according to methods previously reported [25, 26]. The injected doses of ^18^F-DPA-714 and ^18^F-FP-CIT were 38.6 ± 0.7 MBq and 25.4 ± 0.6 MBq, respectively (*n* = 5 for each group). Static emission scans were obtained for 20 min by using the Inveon PET/CT scanner (Siemens Medical Solutions, Germany), 40 min after the injection through the tail vein [27, 28]. A low-dose CT scan was also performed for the attenuation correction of PET data.

PET images were reconstructed by a three-dimensional order-subset expectation maximization algorithm, with voxel dimensions of 0.78 × 0.78 × 0.80 mm^3^. Then, all the reconstructed ^18^F-DPA-714 and ^18^F-FP-CIT PET images were processed to create radiotracer-specific brain templates through the Small Animal Molecular Imaging Toolbox (SAMIT) software package (http://mic-umcg.github.io/samit/). After spatial normalization to the home-made templates, PET images were coregistered to the MRI template embedded in SAMIT. Volumes of interest (VOIs) for the substantia nigra (SN) and striatum (Str) were automatically segmented via the corresponding labeled 3D Atlas, and the averaged standard uptake value (SUV) was calculated.

### 2.4. ELISA Experiments

Blood was collected by cutting the tail tips of rats at 4 weeks after surgery (*n* = 5 for each group). The serum *β*-hydroxybutyrate (BHB) levels were determined using a metabolism assay kit (MAK041, Sigma-Aldrich). Then, transcardial perfusion was conducted with a heparinized-PBS solution. SN of these rats was quickly isolated and washed using ice-cold PBS. After being dried, weighed, grinded, homogenized, and centrifuged, the obtained supernatant was analyzed to determine the levels of tumor necrosis factor-*α* (TNF-*α*, RAB0480, Sigma-Aldrich), interleukin-1*β* (IL-1*β*, AB1832P, Sigma-Aldrich), and interleukin-6 (IL-6, RAB0311, Sigma-Aldrich) in SN through ELISA kits.

### 2.5. Immunohistochemistry and Immunofluorescence

On the day following the PET scan, the rats under anesthesia state were perfused intracardially with cold 4% paraformaldehyde. According the routine immunohistochemistry procedure as previously reported [29], the primary antibodies of anti-TH (1 : 1000; ab184451, Abcam) and anti-DAT (1 : 300; ab184451, Abcam) were used in the current study. The number of TH/DAT-positive cells in the SN was counted, and the integrated density of DAT-positive fibers in Str was assessed with the Imaging J threshold plugin (Image J, NIH/USA) for each hemisphere.

Immunofluorescent staining was performed with the primary antibodies against mGluR5 (1 : 200, ab76316, Abcam), TSPO (1 : 100, ab154878, Abcam), ionized calcium–binding adaptor molecule-1 (Iba-1) for microglia cells (1 : 100, Ab5076, Abcam), and DAPI for nuclei (1 : 5000, sc-3598, Santa Cruz). The mGluR5 positive or TSPO positive microglia cell numbers in SN per square millimeter were counted separately. The ratio of the lesioned (left) to the intact (right) side was analyzed and utilized for the further statistical analysis of both the immunohistochemical and immunofluorescent staining.

### 2.6. Western Blot Analysis

The brain tissue of SN was separated after decapitation, as described in the section of ELISA Experiments (*n* = 5 for each group). Western blot analyses were conducted with the primary antibodies against mGluR5 (1 : 1000, Ab76316, Abcam), p-Akt (1 : 1000, 9271, CST), Akt (1 : 1000, 9272, CST), p-GSK-3*β* (1 : 1000, 9336, CST), GSK-3*β* (1 : 1000, Ab227208, Abcam), p-CREB (1 : 1000, 9198, CST), CREB (1 : 1000, 9197, CST), and *β*-Tubulin (1 : 500, Ab6046, Abcam), following the similar procedure reported previously [23].

### 2.7. Quantitative Reverse Transcription-PCR (qRT-PCR) for mGluR5 mRNA Expression

Total RNA in SN was extracted using the QIAGEN RNeasy kit (*n* = 5 for each group). After being purified and treated with DNase, the mRNA was reverse-transcribed to synthesize the cDNA. The expression level of *mGluR5* mRNA was estimated by qRT-PCR in StepOne™ Real-Time PCR System (Applied Biosystems, USA). The following primers for mGluR5 were used in the process of PCR: F: 5′-AGCTCAACTCCATGATGTTGT-3′ and R: 5′-ATCTCTGCGAAGGTCGTCAT-3′. The quantification of glyceraldehyde-3-phosphate dehydrogenase (GAPDH) expression was employed as the internal control: F: 5′-GAGGCCGGTGCTGAGATTGT-3′ and R: 5′-GGTGGCAGTGATGGCATGGA-3′. The fold change in mRNA levels over control values was computed through the delta-delta method [30].

### 2.8. Chromatin Immunoprecipitation (ChIP) and qRT-PCR for Histone H3K9 Acetylation

The frozen tissue of SN was sectioned and cross-linked in formaldehyde (*n* = 5 for each group). The ChIP procedure was conducted by using the SimpleChIP® Enzymatic Chromatin IP Kit (9003, CST). The collected supernatant was immunoprecipitated with the antibody against H3 acetylation on Lys9 (aceH3K9, 9649, CST), anti-RNA polymerase II (positive control), and normal mouse IgG (negative control). Then, the isolated DNA-histone complex was incubated and treated with RNase A and proteinase K. The DNA associated with aceH3K9 was purified and quantified by qRT-PCR. The aceH3K9 level in the GAPDH promoter region was also studied to establish the specific changes. The fold changes of aceH3K9 level in *mGluR5* promoter region over control were calculated through the delta-delta method [31].

### 2.9. Statistical Analysis

Values were shown as mean ± SEM. All data were analyzed using the SPSS software (IBM SPSS Statistics, Version 25.0). Comparisons among multiple groups were performed by one-way analysis of variance and followed by post hoc Bonferroni test. *P* value less than 0.05 (*P* < 0.05) was considered statistically significant.

## 3. Results

### 3.1. Dopamine Deficient Behaviors

**Figure 1:**
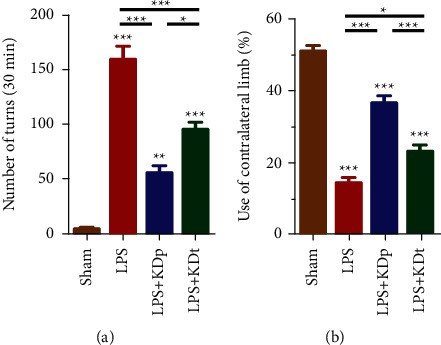
Dopamine deficient behavior test results of all groups. Data were presented as the mean ± SEM in APO-induced rotation test (a) and cylinder test (b). ^*∗∗∗*^*P* < 0.001; ^*∗∗*^*P* < 0.01; ^*∗*^*P* < 0.05; LPS, lipopolysaccharide; KDp, preventive intervention with KD prior to the PD onset; KDt, therapeutic intervention with KD after the PD onset.

Figure 1(a) shows the LPS-induced significant rotational behavior compared with sham group in APO-induced rotation test (159.4 ± 12.2 vs. 4.2 ± 1.5, *P* < 0.001). In addition, both KDp (55.6 ± 6.5) and KDt (95.2 ± 6.4) significantly reduced the numbers of turns seen in the LPS group (both *P* < 0.001). However, compared with KDt group, a significant decrease in the rotation numbers was found in KDp group (*P* < 0.05).

The results of cylinder test illustrated in Figure 1(b) were consistent with those of APO-induced rotation test. Significant contralateral forelimb akinesia was induced by LPS, compared with sham group (14.5 ± 1.4 vs. 51.1 ± 1.5, *P* < 0.001). Both KDp (36.4 ± 2.2, *P* < 0.001) and KDt (23.2 ± 1.7, *P* < 0.05) significantly increased the percentage of using contralateral forelimb seen in the LPS group. However, compared with KDt group, a significant increase in the percentage of using contralateral limb was found in KDp group (*P* < 0.001).

### 3.2. Dual-Tracer PET Imaging

**Figure 2:**
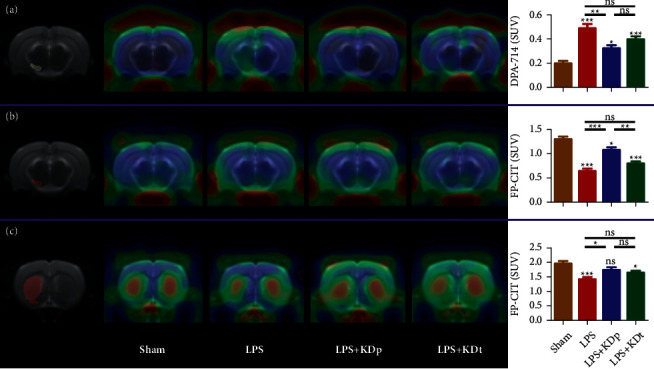
Brain TSPO and DAT PET imaging using ^18^F-DPA-714 and ^18^F-FP-CIT. The SUVs of ^18^F-DPA-714 (a) and ^18^F-FP-CIT (b) in substantia nigra, and those of ^18^F-FP-CIT in striatum (c) were compared among different groups. Data were presented as the mean ± SEM. ^*∗∗∗*^*P* < 0.001; ^*∗∗*^*P* < 0.01; ^*∗*^*P* < 0.05; LPS, lipopolysaccharide; KDp, preventive intervention with KD prior to the PD onset; KDt, therapeutic intervention with KD after the PD onset.

Representative ^18^F-DPA-714 and ^18^F-FP-CIT PET images were displayed in Figure 2. As shown in Figure 2(a), significantly increased SUV of ^18^F-DPA-714 in SN was found in LPS-treated rats (0.487 ± 0.038, 0.324 ± 0.024 and 0.398 ± 0.023 for LPS, LPS + KDp and LPS + KDt groups, respectively) compared with that of sham rats (0.198 ± 0.018, all *P* < 0.05). The intervention of KDp (*P* < 0.01), rather than KDt (*P* > 0.05), significantly reduced the degree of ^18^F-DPA-714 uptake seen in LPS group. No significant difference was found in ^18^F-DPA-714 uptake between KDp and KDt interventions (*P* > 0.05).

Compared with that of sham group (1.301 ± 0.047 and 1.973 ± 0.081, respectively; Figures 2(b) and 2(c)), significantly reduced SUVs of ^18^F-FP-CIT in SN and Str were found in LPS (0.641 ± 0.040 and 1.424 ± 0.073, respectively; both *P* < 0.001) and LPS + KDt (0.798 ± 0.039 and 1.659 ± 0.060, respectively; both *P* < 0.05) groups. The intervention of KDp (1.081 ± 0.048 and 1.772 ± 0.075, respectively; both *P* < 0.05), rather than KDt (*P* > 0.05), significantly increased the degree of SN and Str ^18^F-FP-CIT uptakes seen in LPS group. However, the SN ^18^F-FP-CIT uptakes of LPS + KDp group were significantly lower than those of sham group (*P* < 0.05), but higher than those of LPS + KDt group (*P* < 0.01), which was not found in Str (*P* > 0.05).

### 3.3. Serum BHB Level and Proinflammatory Mediators in SN

**Figure 3:**
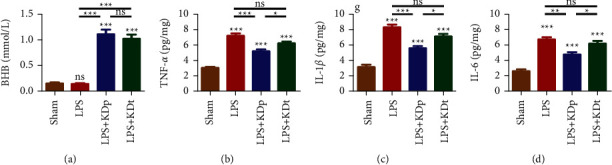
Effects of LPS and KD on the serum BHB level and the production of pro-inflammatory mediators in SN. The level of serum BHB (a), TNF-*α* (b), IL-1*β* (c) and IL-6 (d) were compared among multiple groups. Data were presented as the mean ± SEM. ^*∗∗∗*^*P* < 0.001; ^*∗∗*^*P* < 0.01; ^*∗*^*P* < 0.05; LPS, lipopolysaccharide; KDp, preventive intervention with KD prior to the PD onset; KDt, therapeutic intervention with KD after the PD onset.

Not only the intervention of KDp, but also the KDt significantly increased the serum BHB level (1.116 ± 0.090 and 1.021 ± 0.088 mmol/L, respectively; *P* < 0.001), compared with those of sham and LPS groups with control diet (0.151 ± 0.017 and 0.140 ± 0.016 mmol/L, respectively; Figure 3(a)). No significant difference in serum BHB level was found between KDp and KDt groups (*P* > 0.05).

As shown in Figure 3(b), LPS injection into the SN remarkably upregulated the level of TNF-*α* (7.236 ± 0.287 pg/mg), IL-1*β* (8.320 ± 0.357 pg/mg), and IL-6 (6.706 ± 0.312 pg/mg), compared with sham treatment (3.016 ± 0.144, 3.126 ± 0.314, 2.594 ± 0.253 pg/mg, respectively). The intervention of KDp (5.176 ± 0.249, 5.588 ± 0.283, 4.748 ± 0.324 pg/mg, respectively; all *P* < 0.01), rather than KDt (6.244 ± 0.247, 7.140 ± 0.306, 6.186 ± 0.341 pg/mg, respectively; all *P* > 0.05), significantly suppressed the production of these proinflammatory mediators. Besides, the levels of TNF-*α*, IL-1*β* and IL-6 after the intervention of KDp were significantly lower than those of KDt (all *P* < 0.05).

### 3.4. Deficient Dopaminergic System in SN and Str

**Figure 4:**
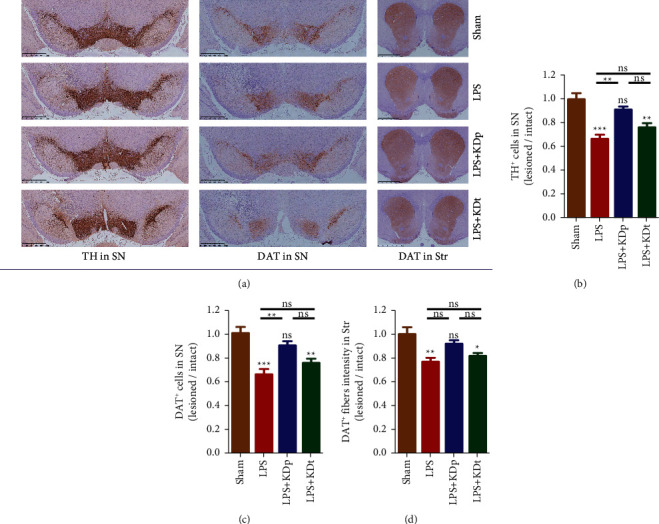
Deficient dopaminergic system in SN and Str. TH^+^ (a), (b) and DAT^+^ (a), (c) nerve cells in SN and DAT^+^ fiber intensity in Str (a), (d) were compared among different groups. Data were presented as the mean ± SEM. ^*∗∗∗*^*P* < 0.001; ^*∗∗*^*P* < 0.01; ^*∗*^*P* < 0.05; LPS, lipopolysaccharide; KDp, preventive intervention with KD prior to the PD onset; KDt, therapeutic intervention with KD after the onset.

The immunohistochemical staining results of TH and DAT were displayed in Figure 4. The numbers of surviving TH^+^ and DAT^+^ nerve cells in SN and DAT^+^ fiber intensity in Str were significantly decreased in rats injected with LPS (0.665 ± 0.035, 0.663 ± 0.047 and 0.770 ± 0.033, respectively; all *P* < 0.01), compared with those with sham treatment (0.996 ± 0.050, 1.009 ± 0.052 and 1.003 ± 0.058, respectively). The intervention of KDp (0.910 ± 0.026 and 0.908 ± 0.033, respectively; both *P* < 0.01), rather than KDt (0.762 ± 0.034 and 0.760 ± 0.035, respectively; *P* > 0.05), significantly inhibited the decline of TH^+^ and DAT^+^ nerve cells in SN seen in the LPS group.

### 3.5. The mGluR5 and TSPO Expression of Microglia Cells in SN

**Figure 5:**
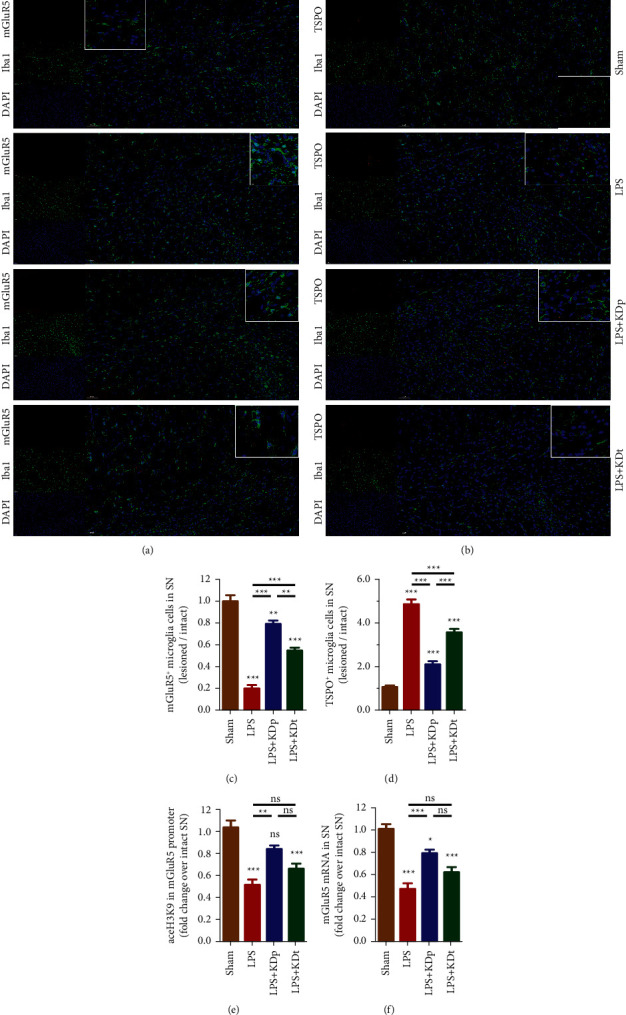
The changes of mGluR5 and TSPO expression induced by LPS and KD in SN. The numbers of mGluR5^+^ microglia cells (a, c), TSPO^+^ microglia cells (b, d), and the levels of aceH3K9 in *mGluR5* promoter region (e) and *mGluR5* mRNA expression (f) were compared among multiple groups. Data were presented as the mean ± SEM. ^*∗∗∗*^*P* < 0.001; ^*∗∗*^*P* < 0.01; ^*∗*^*P* < 0.05; LPS, lipopolysaccharide; KDp, preventive intervention with KD prior to the PD onset; KDt, therapeutic intervention with KD after the PD onset.

Figure 5 shows the LPS-induced significant reduction of mGluR5^+^ microglia cells (0.204 ± 0.029 vs. 1.005 ± 0.055; *P* < 0.001; Figures 5(a) and 5(c)), but there is a significant increase in the TSPO^+^ microglia cells (4.860 ± 0.213 vs. 1.064 ± 0.063; *P* < 0.001; Figures 5(b) and 5(d)) in SN, compared with sham treatment. Both interventions of KDp and KDt prevented the inhibitory effect of LPS on mGluR5^+^ microglia cells (0.791 ± 0.032 and 0.547 ± 0.026, respectively; *P* < 0.001) and suppressed the excessive TSPO^+^ microglia cells induced by LPS (2.108 ± 0.136 and 3.578 ± 0.148, respectively; *P* < 0.001). Compared with LPS + KDt group, however, LPS + KDp group had significantly greater number of mGluR5^+^ microglia cells and fewer number of TSPO^+^ microglia cells (*P* < 0.01).

### 3.6. H3K9 Acetylation in the mGluR5 Promoter Region and mGluR5 mRNA Expression

The levels of aceH3K9 in *mGluR5* promoter region and *mGluR5* mRNA expression were further estimated (Figures 5(e) and 5(f)). The LPS led to a reduction of H3K9 acetylation in the *mGluR5* promoter region (0.519 ± 0.047 vs. 1.037 ± 0.061; *P* < 0.001) and *mGluR5* mRNA expression (0.467 ± 0.051 vs. 1.010 ± 0.041; *P* < 0.001), compared with sham treatment. The intervention of KDp (0.833 ± 0.041 and 0.791 ± 0.031, respectively; both *P* < 0.01), but not KDt (0.666 ± 0.046 and 0.619 ± 0.044, respectively; *P* > 0.05), significantly inhibited the decline of aceH3K9 in *mGluR5* promoter region and *mGluR5* mRNA expression in SN seen in the LPS group. The LPS + KDp group tended to have greater levels of H3K9 acetylation and *mGluR5* mRNA expression than those of the LPS + KDt group, but this difference did not reach statistical significance (*P* > 0.05).

### 3.7. The Expression of mGluR5 and Phosphorylation of Akt/GSK-3*β*/CREB Pathway

**Figure 6:**
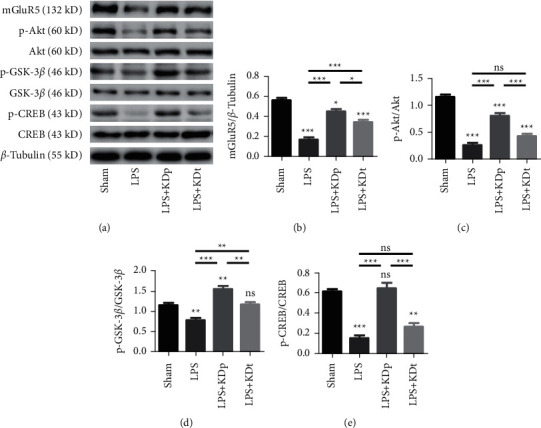
The effects of LPS and KD on the expression of mGluR5, p-Akt, p-GSK-3*β* and p-CREB in SN. The expression of mGluR5 (a, b), and phosphorylation of Akt (a, c), GSK-3*β* (a, d) and CREB (a, e) were compared among multiple groups. Data were presented as the mean ± SEM. ^*∗∗∗*^*P* < 0.001; ^*∗∗*^*P* < 0.01; ^*∗*^*P* < 0.05; LPS, lipopolysaccharide; KDp, preventive intervention with KD prior to the PD onset; KDt, therapeutic intervention with KD after the PD onset.

The expression of mGluR5 (0.167 ± 0.022 vs. 0.560 ± 0.023), p-Akt (0.257 ± 0.047 vs. 1.149 ± 0.059), p-GSK-3*β* (0.772 ± 0.054 vs. 1.137 ± 0.065) and p-CREB (0.148 ± 0.030 vs. 0.580 ± 0.049) in SN was suppressed by LPS, compared with sham group (all *P* < 0.01, Figure 6). The intervention of KDp prevented the downregulation of mGluR5 (0.448 ± 0.024), p-Akt (0.809 ± 0.047), p-GSK-3*β* (1.544 ± 0.075), and p-CREB (0.644 ± 0.056) induced by LPS (all *P* < 0.001). However, the intervention of KDt only prevented the downregulation of mGluR5 (0.336 ± 0.028) and p-GSK-3*β* (1.159 ± 0.071), but not that of p-Akt (0.431 ± 0.041) and p-CREB (0.261 ± 0.046) induced by LPS. The expression of mGluR5 and phosphorylation of p-Akt, p-GSK-3*β*, and p-CREB in LPS + KDp group were significantly higher than those in LPS + KDt group (all *P* < 0.05).

## 4. Discussion

The findings in the current study revealed that LPS caused significant dopamine deficient behaviors and dopaminergic neurons loss, accompanied with neuroinflammation relevant to microglial activation in SN. Dietary intervention with KD suppressed the inflammatory response and exerted neuroprotective effects on LPS-induced rat PD model, via modulating the Akt/GSK-3*β*/CREB signaling pathway mediated by the histone acetylation of *mGluR5* gene promotor region. The prominent finding was that the neuroprotective effect of KDp should be better than that of KDt.

We suggested that utilizing KD with the prevention strategy prior to the PD onset might be more effective than those receiving KD after the onset. It has been noted that LPS-induced loss of dopaminergic neurons in the SN was time-dependent [32]. Moreover, the impairment on the nigrostriatal DA neurons indirectly via microglial activation was permanent [33]. Currently, the limited therapeutic means are mainly used to relieve symptoms and delay the disease progression to some extent. Due to the nonregeneration of neurons loss in neurodegenerative disease, rare clinical therapeutic options exist for reversing its progression [34]. The ketosis induced by KD might require a couple of days to reach a stable state. Therefore, it may be too late to introduce neuroprotective intervention after the onset of irreversible dopaminergic system damage. Initiating the KD or other anti-inflammatory intervention prior to the PD onset in susceptible population should be a more promising strategy.

The findings of current study implied that the anti-inflammatory effects of KD on rat PD model were associated to the modulation of Akt/GSK-3*β*/CREB signaling pathway mediated by mGluR5. Ablation of mGluR5 could stimulate the microglial activation [35], while activating the mGluR5 via selective agonist would attenuate the microglial-induced neurotoxicity [36]. The phospholipase C and protein kinase C signaling pathways were considered to be involved in the anti-inflammatory action of activated mGluR5 [37]. Apart from the degeneration of nigrostriatal dopaminergic neurons, aggregated *α*-synuclein is another prominent PD pathology. Extracellular *α*-synuclein could selectively interacted with mGluR5 in microglia, stimulating the expression level of proinflammatory cytokines [38]. This process was alleviated through the specific agonist of mGluR5 as well. Therefore, targeting mGluR5 is supposed to be an attractive strategy to regulate neuroinflammation for neuroprotection.

The phosphorylation of Akt, also called protein kinase B, is determined by the activation of phosphatidylinositol-3 kinase (PI3K). The activities of multiple substrates in the downstream of PI3K/Akt signaling pathway regulate several physiological or pathophysiological state, including multiple neurodegenerative diseases [39]. Among these substrates, the GSK-3*β* is a serine/threonine kinase too and abundantly expresses in nervous system. Evidence from Alzheimer's disease (AD) animal and cell models suggested that increased activation of this pathway was associated with the improved performance and reduced A*β* levels as well [40, 41]. The neuroprotective mechanism for the anti-inflammatory effects of activated Akt/GSK-3*β* on LPS-induced PD model can be partly attributed to the gene expression inhibition of proinflammatory mediators [29]. Similar to our findings, it has been reported that the downregulation of Akt/GSK-3*β*/CREB signaling would weaken the anti-inflammatory action related to mGluR5 in microglia [42].

Though the exact pathology of PD remains elusive, the oxygen free radicals, trophic factors loss, altered calcium homeostasis, and neuroinflammation have been supposed to be involved [6, 43–45]. However, these pathological processes associated with PD might be alleviated by the multiple neuroprotective mechanism of ketosis induced by KD [46–49]. The anti-inflammatory properties of KD also have been verified in other neurological disorders associated to neuroinflammation, including multiple sclerosis, pain, epilepsy, AD, and spinal cord injury [50–52]. The possible mechanisms supporting its anti-inflammatory actions included the direct inhibition of NLRP3 inflammasome assembly, epigenetic adaptation linked to caloric restriction, polyunsaturated fatty acids, ROS reduction, and gut microbiome [53–58]. Among them, the function of microglial cells can be modulated via various epigenetic mechanisms, such as DNA methylation and histones acetylation. [59]. Ketone body serves as not only an energy substrate, but also a signaling molecule. Moreover, BHB has been classified as a specific histone deacetylase inhibitor as well [22, 60]. Our results further suggested that anti-inflammatory effects of KD were associated to the upregulation of mGluR5/Akt/GSK-3*β*/CREB signaling pathway via increasing the histone acetylation level of *mGluR5* gene promotor region.

Combined with previous studies, our data provide further insight that targeting the mGluR5 with epigenetic modulation would be an attractive strategy to alleviate the microglia activation in PD. Beyond the radiotracers for TSPO and DAT utilized in the current research, the metabolism of ketone body, the function of mGluR5, and the process of epigenetic modification could also be assessed by using corresponding positron agents [61–63]. PET imaging allows the noninvasive detection and monitoring for the whole process of KD exerting anti-inflammatory effects on PD via mediating the histone acetylation or DNA methylation of *mGluR5* gene.

LPS-induced PD model was introduced in our study. However, this inflammatory-predominant model does not fully represent the complete pathological process of PD, so it is uncertain whether our conclusions could be applicable in other PD models. Besides, the *in vivo* and *in vitro* assessments were only conducted at 4 weeks after the LPS injection. Further, longer follow-up would help determine the exact difference in neuroprotection between the KDp and KDt schemes. An alternative to the dietary intervention of KD is the oral administration of ketone body esters, which provide a safe and convenient method for raising plasma ketone body level [64]. However, rather than merely boosting the ketone body level, the neuroprotective mechanisms of KD are diverse, such as caloric restriction or altered gut microbiome. The difference in anti-inflammation between rigorous KD and ketone body esters should be taken into consideration, underlining the necessity for further comparative investigation [19].

## 5. Conclusions

The anti-inflammatory effects of KD on LPS-induced rat PD model were associated to the modulation of Akt/GSK-3*β*/CREB signaling pathway mediated by the histone acetylation of *mGluR5* gene promotor in SN. Dietary intervention with KDp, rather than KDt, should be employed prior to the PD onset in susceptible population, to achieve a more favorable outcome.

## Data Availability

The data generated during the current study are available from the corresponding author upon reasonable request.
